# Pharmacokinetics of Remifentanil: a three-compartmental modeling approach

**Published:** 2013-09-02

**Authors:** Sara Cascone, Gaetano Lamberti, Giuseppe Titomanlio, Ornella Piazza

**Affiliations:** 1 Dipartimento di Ingegneria Industriale, Università di Salerno, Fisciano, Italy; 2 Dipartimento di Medicina e Chirurgia, Università di Salerno, Fisciano, Italy

**Keywords:** Remifentanil, pharmacokinetics, three-compartmental model

## Abstract

Remifentanil is a new opioid derivative drug characterized by a fast onset and by a short time of action, since it is rapidly degraded by esterases in blood and other tissues. Its pharmacokinetic and pharmacodynamics properties make remifentanil a very interesting molecule in the field of 0anesthesia. However a complete and versatile pharmacokinetic description of remifentanil still lacks. In this work a three-compartmental model has been developed to describe the pharmacokinetics of remifentanil both in the case in which it is administered by intravenous constant-rate infusion and by bolus injection. The model curves have been compared with experimental data published in scientific papers and the model parameters have been optimized to describe both ways of administration. The *ad hoc* model is adaptable and potentially useful for predictive purposes.

## I. INTRODUCTION

**Fig 1:**
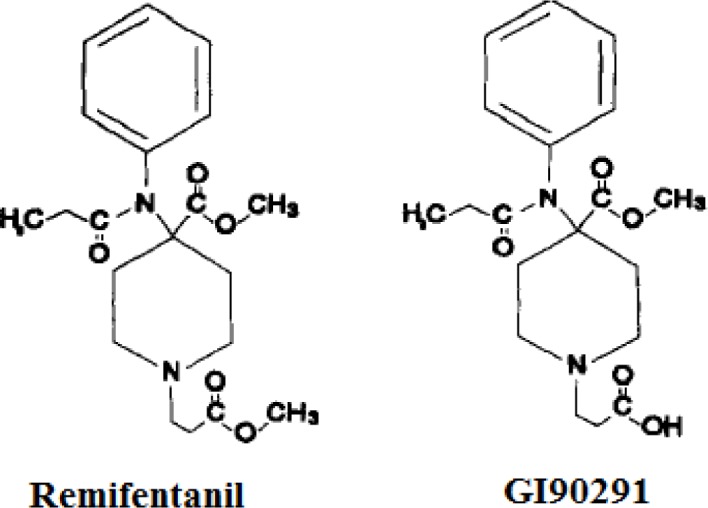
Chemical structures of remifentanil and its metabolite.

The topic of the development of new opioid anesthetic agents is mainly to increase potency, and reduce the cardiovascular toxicity. For this purposes, recently a new kind of opioid derivative drug, remifentanil, has been synthetized. Remifentanil is an ultra-short acting opioid and it is subjected to metabolism by esterases in blood and other tissues. *In vivo* studies demonstrated an extensive metabolism of this drug by ester hydrolysis [ 1 ] . The primary metabolic pathway experienced by remifentanil is the formation of a carboxylic acid metabolite (named GI90291) obtained by de-esterification. The chemical structures of remifentanil and its primary metabolite are shown in Fig 1 . It has been demonstrated on animals [ 1 ] that the pharmacodynamics of remifentanil is very similar to the other opioid drugs, this fact, combined with the reduced effects on the cardiovascular system makes the use of remifentanil very attractive in anesthesia. Remifentanil is generally administrated by the intravenous route. Because of the very short half-life of the drug, usually a bolus injection is administered to raise the blood concentration immediately, then a slower intravenous infusion is used to maintain the effective plasma concentration. Even if it is recommended to infuse remifentanil only during general anaesthesia procedures, the single/repeated bolus injections could be used in clinical situation in which a brief period of intense analgesia is required and the set - up of a continuous infusion pump is difficult (i.e. painful diagnostic and therapeutic procedures outside the operating theater). For this reason, it is particularly interesting to model what happen in the plasma concentration of remifentanil after the bolus injection or continuous infusion administration. Different kinds of models have been proposed, the simplest of which is the compartmental one [ 2 ] , alternatively, the physiologically based approach [ 3 ] is more complex and potentially more exhaustive.

The aim of the present work is to develop and validate a new simple model using the compartmental modeling approach to evaluate the remifentanil plasma concentration in case of bolus injection and continuous infusion.

## II. MODELING

**Fig 2:**
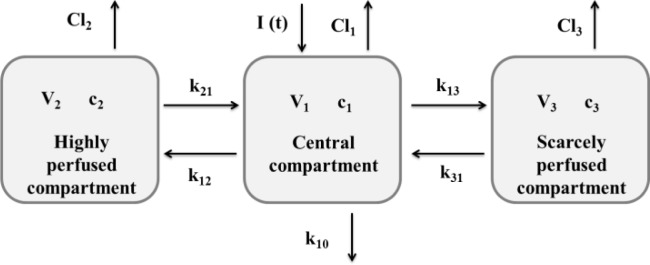
A schematic of the three-compartmental model.

In the three-compartmental modeling, three compartments describe the fate of a drug once administered: the central compartment, which represents the plasma; the highly perfused compartment, which represents the organs and tissues highly perfused by the blood; and the scarcely perfused compartment, which represents the organs and tissues scarcely perfused by blood. A schematic of the model is shown in Fig 2 .

To model the pharmacokinetic of remifentanil, the case of intravenous infusion has been studied. Thus, an amount of drug has been evaluated as inlet in the central compartment. The processes which cause the variation of the plasma concentration are: the absorption, the distribution, and the excretion of the drug. These phenomena have to be taken into account in the modeling. *I(t)* is defined as the function which describes the drug introduction by intravenous infusion (which could be an infusion at constant rate of administration or a bolus) in the central compartment. The drug concentration in the compartments could be evaluated solving the mass balance in the compartments, which could be written as:

Central compartment:

$$\begin{array}{l} V_{1}\cdot \frac{dC_{p}}{dt}=-Cl_{1}\cdot C_{1}+k_{21}\cdot V_{2}\cdot C_{2}+k_{31}\cdot C_{3}\cdot V_{3}+\\ \;\;\;\;\;\;\;\;\;\;\;\;\;\;\;\;\;\;\;\;\;-\left[\left(k_{12}+k_{13}+k_{10}\right)\cdot C_{1}\right]\cdot V_{1}+I(t) \end{array}$$

Highly perfused compartment:

$$V_{2}\cdot \frac{dC_{2}}{dt}=k_{12}\cdot C_{1}\cdot V_{1}-k_{21}\cdot C_{2}\cdot V_{2}-Cl_{2}\cdot C_{2}$$

Scarcely perfused compartment:

$$V_{3}\cdot \frac{dC_{3}}{dt}=k_{13}\cdot C_{1}\cdot V_{1}-k_{31}\cdot C_{3}\cdot V_{3}-Cl_{3}\cdot C_{3}$$

In which, *C _P_* , *C _2_* , and *C _3_* are, respectively, the drug concentrations of the central, highly perfused, and scarcely perfused compartments. *V _1_* , *V _2_* , and *V _3_* are, respectively, the volumes of the central, highly perfused, and scarcely perfused compartments. *Cl _1_* , *Cl _2_* , and *Cl _3_* are, respectively, the clearances (rates of drug elimination) of the central, highly perfused, and scarcely perfused compartments. *k _12_* and *k _21_* are the transport coefficients between the central and the highly perfused compartments; *k _13_* and *k _31_* are the transport coefficients between the central and the scarcely perfused compartments. Finally, *k _10_* is the kinetic constant of drug elimination from the central compartment. The kinetics of elimination and transport between the compartments have been considered first order kinetics. These equations have to be solved coupled with their initial conditions:

$$\{\begin{array}{l} @t=0,C_{P}=0\\ @t=0,C_{2}=0\\ @t=0,C_{3}=0 \end{array}$$

The three equations are inter-dependent, thus, they have to be solved simultaneously to evaluate the drug concentration in all the compartments.

Once identified the transport phenomena which take place in the compartments and defined the differential equations able to solve the mass balances in the compartments, the value of the parameters has to be evaluated. To evaluate the parameters value, the model has been used to fit the experimental data taken in literature [ 4 , 5 ] , which refer both to intravenous infusion and bolus. Defining an error between the experimental data and the model evaluation:

$$ɛ(\underline{p})=\frac{1}{n}\cdot \sum_{i=1}^{n}\frac{\left|\text{log}C_{Pi}-\text{log}C_{Pm}\left(t_{i},\underline{p}\right)\right|}{\text{log}C_{Pi}}$$

In which *n* is the number of experimental data for each experiment, *C _Pi_* − *C _Pm_* ( *t _i_* , ***p*** ) is the difference between the experimental plasma concentration and the model prediction at the time *t _i_* , ***p*** being the vector of the model parameters. Minimizing this function it is possible to find the values of the parameters which better approach the experimental data.

The set of three ODEs constituting the model have been solved by a code developed using Matlab, and the same software was used to find the value of ***p*** minimizing the function ɛ( ***p*** ).

## III. RESULTS

**Fig 3:**
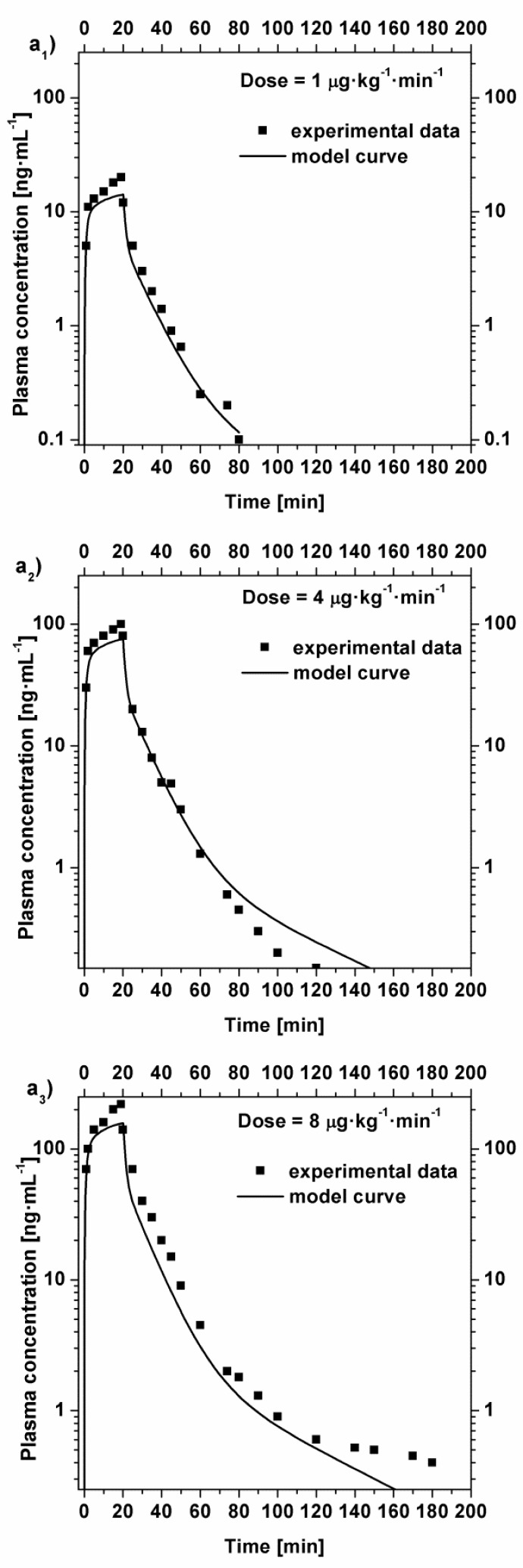
Comparison between the experimental plasma concentration value [ 5 ] and the model curves in the case of intravenous constant-rate infusion with an infusion time of 20 minutes. a _1_ ) plasma concentration after a dose of 1 μg·kg ^−1^ ·min ^−1^ ; a _2_ ) plasma concentration after a dose of 4 μg·kg ^−1^ ·min ^−1^ ; a _3_ ) plasma concentration after a dose of 8 μg·kg ^−1^ ·min ^−1^ .

The model simulations obtained are shown in Fig 3 and compared with the experimental data [ 5 ] in the case of intravenous constant-rate infusion. Each curve has been obtained as average values due to the administration to two subjects. During the 20 minutes infusion, a total of 14 blood samples were taken. After stopping the infusion, 16 blood samples were taken, up to 240 min after the stop. Therefore, each history was described by 30 sample data.

As could be seen from the graphs, the model reproduces satisfactorily the experimental data. In particular, the plasma concentration following the administration of a dose of 1 μg·kg ^−1^ ·min ^−1^ is well approximated, however, the concentration of higher doses (4 and 8 μg·kg ^−1^ ·min ^−1^ ) are not well approximated for periods longer than 90 minutes, this is probably due to the fact that the supposed elimination kinetic could be still optimized.

**Fig 4:**
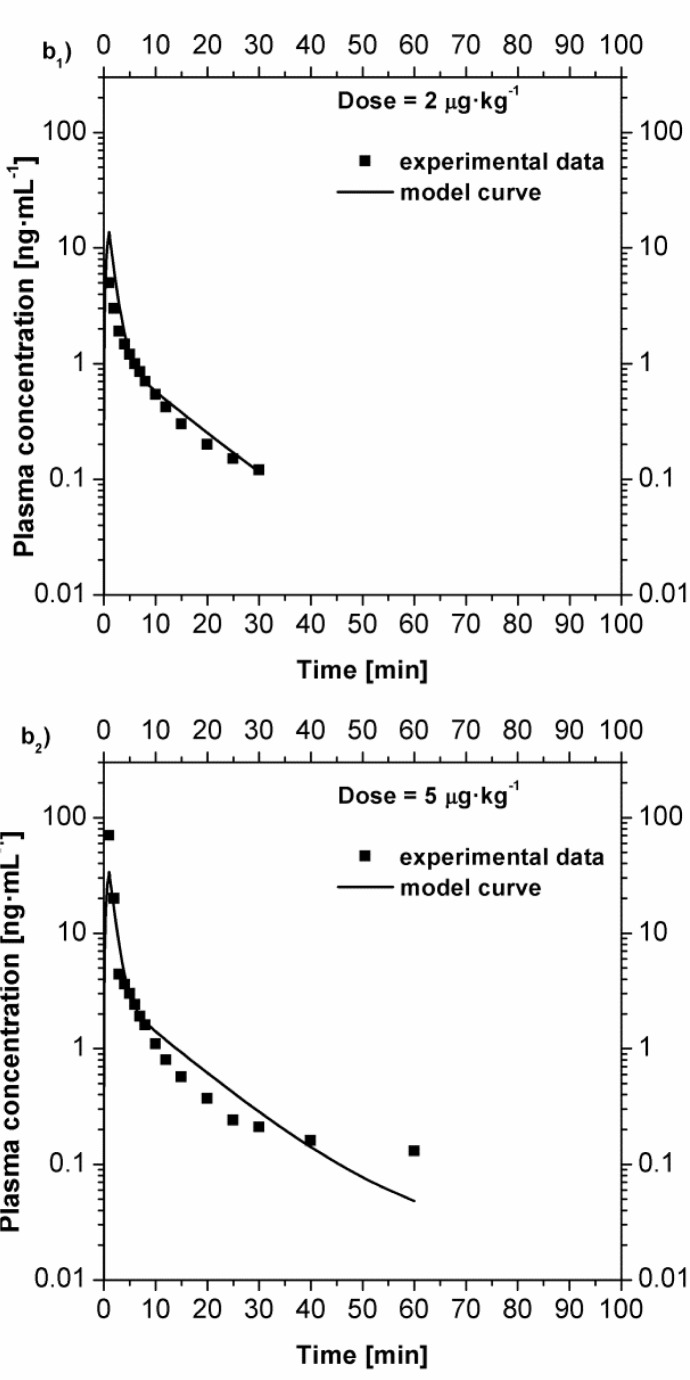

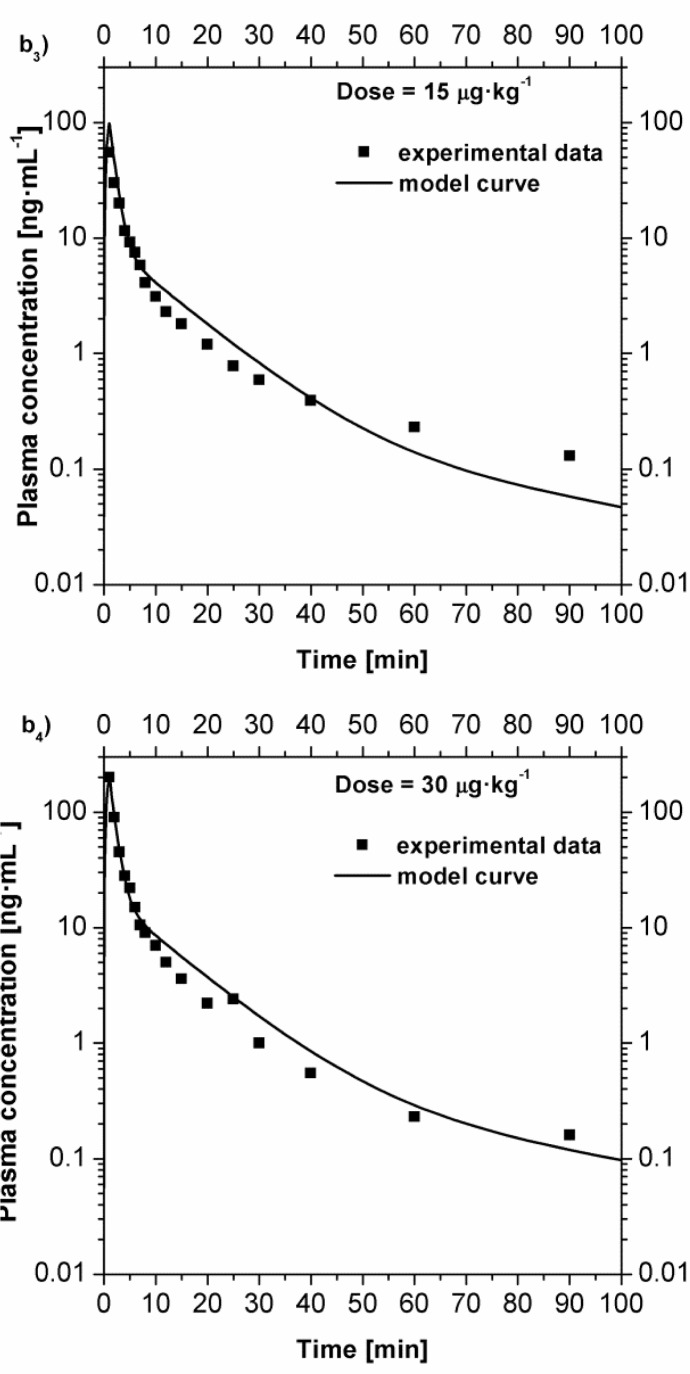
Comparison between the experimental plasma concentration value [ 4 ] and the model curves in the case of fast intravenous infusion (bolus). b _1_ ) plasma concentration after a dose of 2 μg·kg ^−1^ ; b _2_ ) plasma concentration after a dose of 5 μg·kg ^−1^ ; Comparison between the experimental plasma concentration value [ 4 ] and the model curves in the case of fast intravenous infusion (bolus). b _3_ ) plasma concentration after a dose of 15 μg·kg ^−1^ ; b _4_ ) plasma concentration after a dose of 30 μg·kg ^−1^ .

Furthermore, the same model has been used to reproduce the remifentanil plasma concentrations in the case of bolus injection and compared with the experimental data [ 4 ] in Fig 4 . In this case the administration has been evaluated as a fast infusion (bolus), thus the plasma concentration immediately rise to a high value. Each curve has been obtained as the average value over six patients (three men and three women). Over 360 minutes, 21 blood samples were collected and assayed for remifentanil. Once again, the model curves satisfactorily approximate the experimental data.

The values of the model parameters obtained after the optimization routine, are shown in Table 1 . The model developed has been used to evaluate the plasma concentration both in the case of intravenous constant-rate infusion and intravenous bolus, which has been reproduced simulating a very fast infusion in the central compartment. This is a remarkable improvement to the compartmental modeling: in fact, once the model parameters have been evaluated for a certain administration, the model is able to predict the drug plasma concentration varying not only the dose, but also the infusion rate of the drug.

## IV. CONCLUSIONS

In this work a three-compartmental model has been developed to reproduce the evolution of remifentanil plasma concentrations after intravenous constant-rate infusion and intravenous bolus. The main phenomena of absorption, distribution, and metabolism have been identified and the mass balances for the three compartments have been written. The model has been then used to reproduce plasma concentrations taken from literature and the best values of the model parameters have been found minimizing the error between model curves and experimental data.

Several studies have been conducted to develop a model which is able to reproduce the remifentanil pharmacokinetics. The aim of these studies is to compare the measured pharmacokinetic features of remifentanil after an intravenous infusion to the model prediction [ 6 ] . In particular, the compartmental analysis has been extensively used and compared with the experimental data, taken after intravenous infusion [ 5 ] or bolus [ 4 ] . Blood concentration and time data after a computer-controlled infusion of remifentanil could be analyzed by nonlinear regression using the NONMEM program (University of California) [ 7 ] which may produce predicted and individually predicted values (post hoc Bayesian estimates). The initial two-stage analysis comparing one-, two-, and three-compartmental models found that the two-compartmental model shows the best fit to the experimental data. This result was also confirmed by a population analysis. A more complex analysis of both pharmacokinetics and pharmacodynamics of remifentanil has been also approached [ 8 ] . The pharmacokinetic/pharmacodynamic relationship has been evaluated using non-linear regression analysis. The pharmacokinetics have been described using a one-compartment intravenous infusion model. Moreover, the pharmacodynamics have been fitted using inhibitory model. A statistical evaluation of the goodness of the models has been carried out following the Akaike Information Criterion [ 9 ] . According to this analysis, the decrease of the SSE using a model with a large number of parameters is useful if and only if it overcomes the increase in the number of parameters with respect to the use of a model with a limited number of parameters. In this case, the simple model has shown the best overall fitting results.

Nevertheless, once the value of the parameters has been evaluated, our simple model was able to describe the remifentanil concentration on blood for different ways of administration. This is a remarkable improvement to the compartimental modelling: in fact once the model parameters have been evaluated for a certain kind of administration, the model is able to predict the drug plasma concentration varying not only the dose but also the infusion rate of the drug. This feature makes the model more versatile than the other available in literature and very useful for predictive purposes.

## Figures and Tables

**TABLE I:** Values and dimensions of the three-compartmental model parameters.

** Parameter **	** Optimized value **	** Parameter **	** Optimized value **
* V _ 1 _*	7.88 mL	* k *_ 10 _	0.172 min ^ −1 ^
* V _ 2 _*	23.9 mL	* k *_ 12 _	0.373 min ^ −1 ^
* V _ 3 _*	13.8 mL	* k *_ 21 _	0.103 min ^ −1 ^
* C _ l1 _*	2.08 mL·min ^ −1 ^	* k *_ 13 _	0.0367 min ^ −1 ^
* C _ l2 _*	0.828 mL·min ^ −1 ^	* k *_ 31 _	0.0124 min ^ −1 ^
* C _ l3 _*	0.0784 mL·min ^ −1 ^		
